# Protistan and fungal diversity in soils and freshwater lakes are substantially different

**DOI:** 10.1038/s41598-020-77045-7

**Published:** 2020-11-18

**Authors:** G. Sieber, D. Beisser, C. Bock, J. Boenigk

**Affiliations:** Department of Biodiversity, University of Duisburg-Essen, Essen, Germany

**Keywords:** Microbial communities, Molecular biology

## Abstract

Freshwater and soil habitats hold rich microbial communities. Here we address commonalities and differences between both habitat types. While freshwater and soil habitats differ considerably in habitat characteristics organismic exchange may be high and microbial communities may even be inoculated by organisms from the respective other habitat. We analyze diversity pattern and the overlap of taxa of eukaryotic microbial communities in freshwater and soil based on Illumina HiSeq high-throughput sequencing of the amplicon V9 diversity. We analyzed corresponding freshwater and soil samples from 30 locations, i.e. samples from different lakes across Germany and soil samples from the respective catchment areas. Aside from principle differences in the community composition of soils and freshwater, in particular with respect to the relative contribution of fungi and algae, soil habitats have a higher richness. Nevertheless, community similarity between different soil sites is considerably lower as compared to the similarity between different freshwater sites. We show that the overlap of organisms co-occurring in freshwater and soil habitats is surprisingly low. Even though closely related taxa occur in both habitats distinct OTUs were mostly habitat–specific and most OTUs occur exclusively in either soil or freshwater. The distribution pattern of the few co-occurring lineages indicates that even most of these are presumably rather habitat-specific. Their presence in both habitat types seems to be based on a stochastic drift of particularly abundant but habitat-specific taxa rather than on established populations in both types of habitats.

## Introduction

Despite the central importance of protists and fungi at the basis of soil and aquatic food webs comparative analyses of protists and fungi community composition in soils and freshwaters are rare. In both habitats protists are a very diverse and ubiquitously distributed group of organisms. They fulfill important ecosystem functions^1–3^ channel bacterial secondary production from the microbial food web to higher trophic levels^4,5^ thereby interacting indirectly and directly with other taxa such as fungi^6,7^. Particularly in aquatic habitats they are also the dominant primary producers^8^. But constraints structuring their diversity differ fundamentally between both habitats: for instance, freshwater habitats are more homogeneous than soil habitats due to mixing of the water body^9,10^, the availability of water in soils is constrained by e.g. evaporation and soil irrigation^11–13^, and soils are more heterogeneous than aquatic habitats consisting of various microhabitats^14–17^. As diversity is largely sustained and maintained by habitat heterogeneity^18–22^ soils are therefore expected to harbor a higher protists richness even in small volumes^23,24^ as compared to aquatic habitats.

Beyond habitat heterogeneity the distinct habitat properties of soil and water put different demands upon organisms inhabiting these habitats. Microbial organisms are differentially challenged by environmental factors of soil and freshwater habitats^25–28^. Their adaptations may therefore systematically differ eventually leading to exclusive communities (at least in part) of soils and freshwaters. In fact, protist communities in aquatic habitats comprise a high share of phototrophs such as diatoms and green algae while these groups are less important in soil communities^29^. But also the heterotrophic protist communities differ between both habitat types. In soils gliding and substrate attached taxa such as amoeba and cercozoans have a high share^29^. In contrast, free swimming taxa such as katablepharids and (heterotrophic) dinoflagellates as well as the filter-feeding choanoflagellates are more abundant in the pelagial of aquatic habitats^30,31^. However, several studies suggest that both habitats may not be that different for microorganisms. First, even soil pores have been suggested to be basically aqueous environments for microbial organisms and soil protists therefore to be basically aquatic organisms^32–34^: in both habitats protists move by gliding or swimming and they feed by similar mechanisms such as interception feeding, filter feeding and grazing. Secondly, as there is no clear boundary between soil and freshwater habitats organisms may further easily be exchanged between both habitats. Dispersal via passive mechanisms like surface run-offs, interstitial and groundwater flow and flooding is well known^35–38^. Further, active dispersal enhances exchange of organism between different habitats. Microorganisms are dispersed by anthropogenic factors like ballast water, aquaculture, fishing and watersport^39,40^. Resting stages do even survive long distance transport and long transport times^41–43^. Also non-anthropogenic factors such as animals act as vector^44,45^. Thus, due to the similar microhabitat properties with respect to movement and feeding and potentially easy exchange between both habitats they may be inhabited by basically the same protist species^37^. However, easy dispersal does not necessarily result in establishment. Dead organisms and resting stages may wrongly indicate the presence of newly introduced microorganisms. But even microorganisms which survive in the new habitat may be out-competed by adapted/acclimatized taxa. In particular the potential dessication of soil pores may pose environmental constraints selecting against freshwater organisms while soil organisms may miss adaptations for buoyancy required for staying in the euphotic zone.

Thus, although the dominant microbial eukaryotes differ considerably between different habitats^33,44–46^, organisms considered as typical for aquatic environments may occur also in soil (e.g. Choanoflagellata^47^) and vice versa fungi (e.g. soil fungi occur also on submerged material^48^).

Based on the high diversity of microhabitats in soils and the capacity of soils and sediments as seed bank the (active) freshwater communities may represent merely subfractions of the more diverse soil communities^49,50^. These findings suggest an inoculation of the (freshwater) habitats with individual taxa or the existence of habitat–generalistic taxa which occur in both, soil and fresh water. Studies on bacterial and archaeal taxa showed that upslope soil environments contain the core community, which inoculate downslope surface waters (58%, 43% respectively), but only 18% of the upslope eukaryotic microbes were found downslope in the arctic tundra^49–51^. Accordingly, protist freshwater communities may be expected as subsets of protist soil communities possibly further modified by environmental constraints.

Here we address the community overlap between soil and freshwater protists using a geographically consistent set of 30 sampling sites comprising samples from lakes and ponds and adjoined soil samples from the respective catchment areas. We studied the molecular diversity of communities based on Illumina amplicon sequencing of the hypervariable 18S SSU V9 rRNA gene region^3,29,52,53^.

We expect that OTU richness in soils is much higher as in freshwaters^21,54,55^, and we hypothesize that the freshwater communities are to a large extent composed of taxa present also in soils, i.e. a subset of the soil communities, even though a certain fraction of taxa may be habitat-specific^56,57^. Following this idea, we further hypothesize that the community is composed mostly of habitat-generalists, occurring in both habitat types and comprising only few rather habitat-specific organisms^29,58–60^.

However, we expect the relative abundance of taxa to be considerably different between both habitat types.

## Methods

### Sampling and sample processing

We sampled freshwater lakes and corresponding soils from a geographic consistent set in Germany. Site selection focused on natural lakes (and reservoirs) and corresponding soil sites which were typical for the respective area (Fig. S1).

All samples were taken during mid-summer, soil samples were taken 2016 and freshwater samples were taken 2012 (see Table S1 and Fig. S1 for details on sampling sites). Samples were taken years apart, as the idea of the study was to compare freshwater and soil habitats on a similar geographic scale, i.e. use soil samples from the (direct) catchment area of the respective freshwaters, but to reduce effects of short-term cross-contaminations between both habitat types due to flooding, intense rainfall, recently performed watersports, etc. which may have blurred the results. Freshwater sampling and sample processing is described in detail in Boenigk et al. (Genbank, PRJNA414052)^46^. Briefly, the freshwater samples were collected two meters from the waterside and between 0.2 and 0.8 m below the water surface. Freshwater samples were filtered on Isopore 0.2 µm polycarbonate filters (Merck Chemicals GmbH, Darmstadt, Germany) until the filters were clogged (biomass normalized). The filters were air dried and subsequently frozen in liquid nitrogen (Cryoshippers). The filters were stored at − 80 °C in the laboratory until DNA extraction^46^.

Soil samples were taken as top soil composite samples from the upper 5 cm of the surface soil (A horizon) with a distance of around 50 m from the corresponding freshwater lake to avoid collecting samples from the direct floodplain. For each soil sample three subsamples within one square meter were taken and roots, as well as other larger particles like stones and fir needles were manually removed. The three subsamples were combined, mixed thoroughly and filled in 1.5 ml tubes. Samples were immediately preserved in a cryoshipper and stored at − 80 °C until DNA extraction.

Soil DNA was extracted by using the Power Soil DNA Isolation Kit (MoBio, Germany) according to the instructions of the supplier with the following modification: vortexing at maximum speed subsequent centrifugation and transfer of the supernatant to a new tube was repeated until no new pellet was formed. Subseqently, two washing steps with C5 solution (MoBio, Germany) were performed and a final dry centrifugation was conducted two times. For PCR we used the forward primer Euk1391F (5′-GTACACACCGCCCGTC-3′^61,62^) and the reverse primerbased on Bock et al.^63^, i.e. a combination of the primers ITS2_Dino (5′-GCTGCGCCCTTCATCGKTG-3′) and ITS2_broad (5′-GCTGCGTTCTTCATCGWTR-3′) in a ratio of 10%:90%. Primers used for freshwater and soil samples were identical.

The mixture for the PCR of the soil samples consisted of: 0.5 μl DNA template (depending on the concentration dilutions of 1:1, 1:10, 1:50 or 1:100 were used) in 25 μl reactions with 0.25 units Phusion Taq (Thermo Fisher Scientific), 0.75 μM primers, 0.5 μl of 0.4 mM dNTPs and 5× Phusion HF buffer. The PCR-cycling conditions included an initial denaturation step at 98 °C for 3 min followed by 35 cycles each including a denaturation step at 98 °C for 30 s, annealing step at 61 °C for 75 s, and an elongation step at 72 °C for 60 s. The PCR was completed by a final extension step at 72 °C for 10 min.

The quality and quantity of the DNA was checked using a Thermo Scientific NanoDrop ND-2000 UV–Vis spectrophotometer (Thermo Fisher Scientifics), electrophoresis in 1% agarose gel stained with ethidium bromide (0.2 μg mL^−1^) and ImageJ (v. 1.51d)^64^. Equimolar subsamples were pooled and commercially sequenced using paired-end HiSeq 2500 sequencing, applying 2 × 300 bp reads using the “rapid run” mode on the Illumina platform of a sequencing provider (Fasteris, Geneva, CH)^46^.

The sequencing reads are available through the project PRJNA675443.

## Bioinformatical processing

### Sequence filtering

Adapter-, quality trimming and demultiplexing using MID sequences were performed by the sequencing company (Fasteris). The base quality of the sequence reads was checked using FastQC^65^. A split-sample filtering protocol for Illumina amplicon sequencing was used by two technical replicates per DNA sample^62^. The raw sequences were quality filtered (PRINSEQ-lite v.0.20.4)^66^ to remove reads with an average Phred quality score below 25. The paired-end reads were assembled and quality filtered with PANDASeq (v2.10)^67^. All reads with uncalled bases, an assembly quality score below 0.9, a read overlap below 20, or a base with a recalculated Phread-score below 1 were removed. After dereplicating chimeras were identified and filtered using UCHIME (v7.0.1090)^68^ with default settings. Sequences that were not present in both sample branches were discarded^62^. The bioinformatical pipeline is available on github (https://github.com/MW55/Natrix).

### Statistical analyses

Data processing was carried out with R^69^ version 3.6.1. Remaining reads after the filtering were clustered using SWARM (v 2.1.9)^70^, then clustered by identical V9 sequences (first 150 bp, identity = 100%, to remove the ITS1 region from the sequences to obtain OTUs which are based on the V9 region) (“V9_Clust.R” by Jensen 2017 available on https://github.com/manfred-uni-essen/V9-cluster)^71^ and aggregated to OTUs. Taxonomic assignment was done by searching against the NCBI nt database using BLASTn (Dec 05th 2017)^72^ using an 85% identity value for the best hit and adjusting the taxonomy according to Adl et al.^73^. All sequences assigned to Metazoa and Embryophyta were discarded, as protists and fungi are the targets of this study. OTUs that represent less than 0.0005% of a respective site were discarded (total number of reads/OTUs). For habitat comparisons we restricted the analysis to OTUs that occurred in at least two sites. Rarefaction curves were created by the R-package vegan (v2.5.6.)^74^ and samples that did not reach saturation were discarded from further analyses.

True diversities are based on the Shannon index and were computed from the raw OTU table using R-package RAM as well as Pielou’s evenness^75^. True diversity were chosen as a measurement for diversity as it is not a non-linear diversity index (e.g. Shannon index, Simpson index) but is suitable for comparisons. Diversities (true diversities, evenness and richness) were statistically compared using a Mann–Whitney-U-test. For distance decay relationships, we replaced zeroes in our raw dataset based on a Bayesian-multiplicative replacement (cmultRepl, R zCompositions package^76^) and calculated the Aitchison distance, as we are dealing with compositional data^77^. Aitchison distance is used as an community dissimilarity proxy. Linear regression slopes of distance decay relationships were tested against zero with an ANOVA.

Figures were prepared with R (R Core Team) version 3.6.1, CorelDRAW × 8 and ArcGIS Pro 2.6.

## Results

### Differential pattern of diversity between soil and freshwater

Total number of assembled reads after filtering was 35,445,831 which grouped in 33,745 OTUs. Of these 18,745 OTUs (corresponding to 13,957,146 reads) were found exclusively in soil sites and 14,337 (corresponding to 13,429,169 reads) exclusively in freshwater sites. OTU richness was 1212 ± 420 OTUs per sample in soil and 852 ± 427 OTUs per samples in freshwater. For habitat comparisons we further restricted the analyses to OTUs occurring in at least two samples resulting in 10,515 OTUs (34,139,127 reads) with an OTU richness of 918 ± 334 per sample in soil and of 588 ± 290 per sample in freshwater.

**Figure 1:**
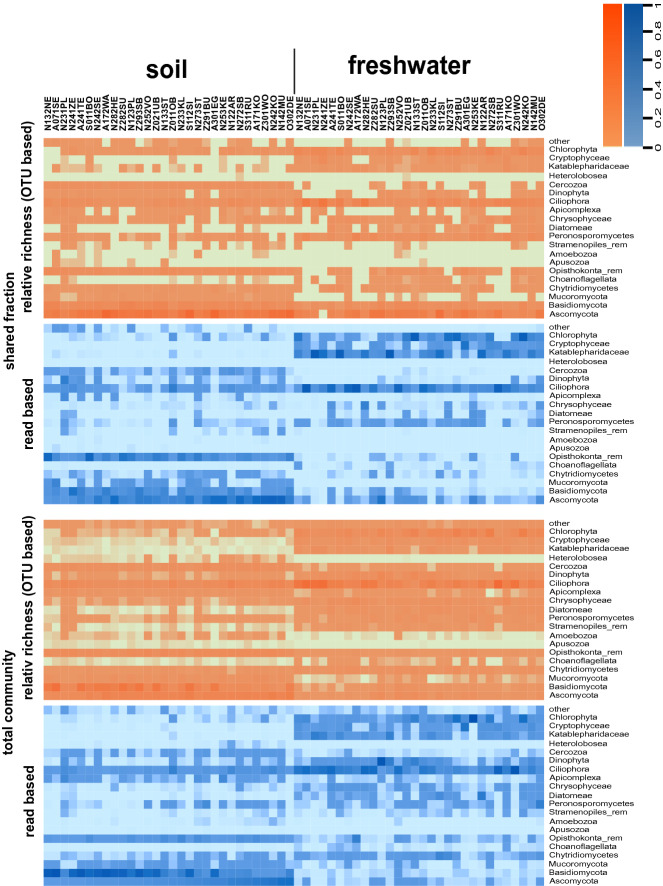
Relative total community composition (bottom) and relative total composition of the overlapping OTUs (top). Taxonomic composition based on relative abundance of OTUs that are present in at least two sites (blue) and taxonomic composition based on presence absence data of OTUs that are present in at least two sites (orange). The sites are sorted by the abundance of Ascomycota in soil sites. Remaining OTUs which could be assigned to a rough taxonomic level were marked as _rem. Created with the R-package gplots^78^.

Community composition and richness strongly differed between soil and freshwater (Fig. 1). In soil Ascomycota, Basidiomycota, remaining Opisthokonta and Ciliophora dominated while Ciliophora and algae dominated freshwater samples, in particular by Chlorophyta and Dinophyta. While in soil 4530 of the 6744 OTUs were affiliated with fungi, in freshwaters only 938 of the 4434 OTUs were affiliated with fungi.

Estimates of the effective number of eukaryotic microbial OTUs (true diversity) revealed that the soil community is more diverse than the freshwater community (p < 0.05, Fig. S2). This was largely due to the higher OTU richness in soil samples while evenness was rather similar between freshwater and soil sites (Fig. S5). When restricting the analysis to either protists or fungi we found different pattern. We found a higher diversity in soil when the analysis was restricted to protists excluding fungi (p < 0.05), even though soil protist richness was lower than freshwater protist richness (Fig. S2). The higher diversity in soils is thus to a large extend related to a higher evenness (Fig. S5) while protist communities in freshwater are rather dominated by individual OTUs. In contrast, the diversity of fungi was similar in freshwater and in soil (p > 0.05) (Fig. S2) but the architecture of fungal community composition differed between soils and freshwater. While richness of fungi was higher in soils, evenness was higher in freshwater resulting in similar diversity indices (Figs. S2, S5).

Corresponding to the effective OTU number we also found that richness was significant higher in soil sites compared to fresh water (p < 0.05). Richness revealed a deviating pattern for protists and for fungi: richness of fungi was significantly higher in soil than in freshwater (p < 0.05) while richness of protists was significantly higher in freshwater (p < 0.05).

Soil did not only hold a higher richness but also a higher dissimilarity among samples as compared to freshwater: The community dissimilarity analyses clearly revealed a generally higher dissimilarity among soil samples (Fig. S3). However, neither soil nor freshwater community dissimilarity changed significantly with increasing distance up to 800 km, i.e. both slopes of the linear regressions slopes are not significantly different from 0 (ANOVA, p < 0.001).

### Community overlap between aquatic and terrestrial habitats

**Figure 2:**
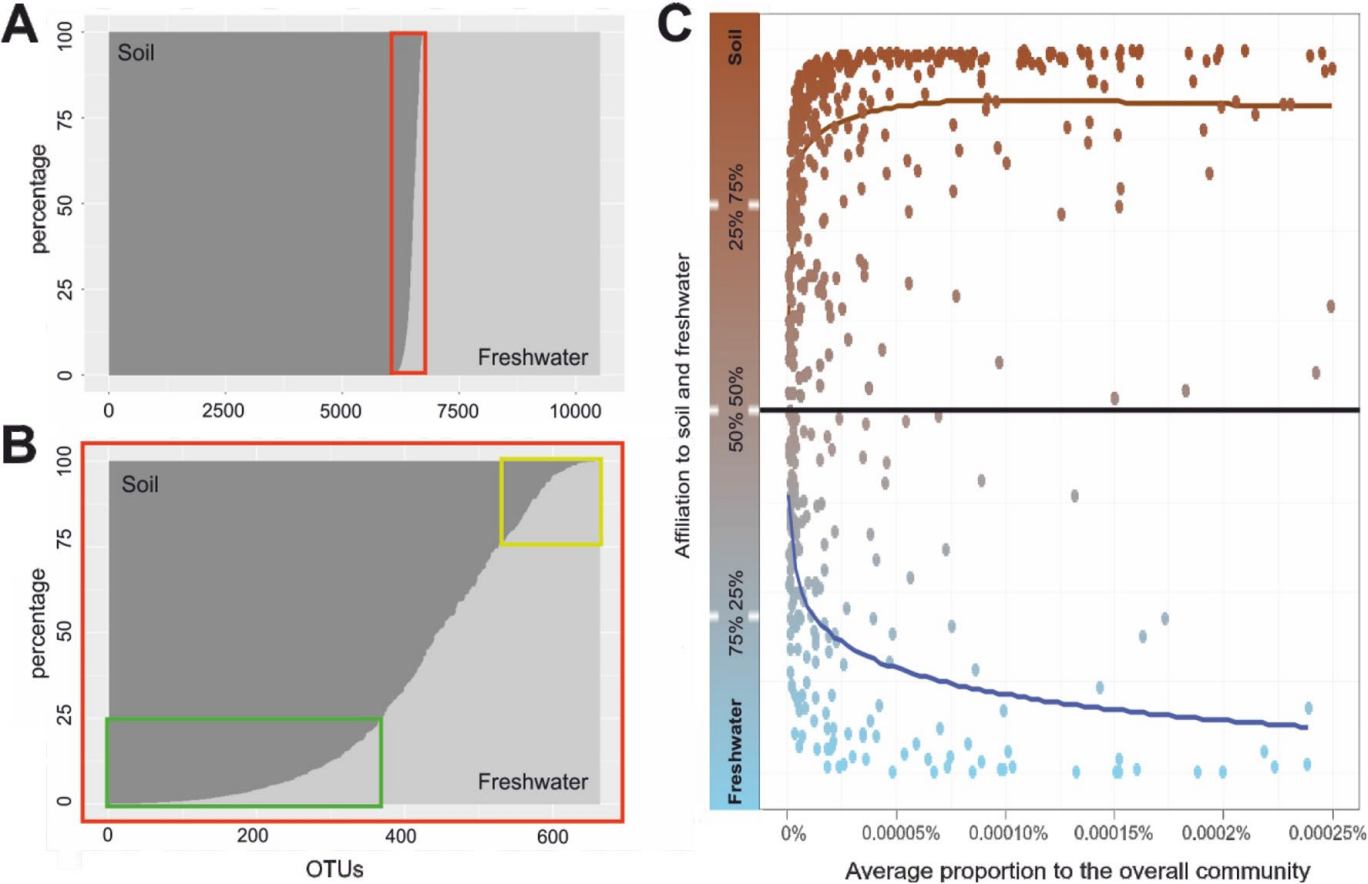
(**A**) Distribution of OTUs across soil (dark) and fresh water (light). The red frame marks overlapping OTUs. (**B**) Is an enlarged view of (**A**), namely OTUs that occur in soil and freshwater (red frame). The green frame indicates OTUs that have their origin rather in soil and the yellow frame indicates OTUs that have their origin rather in freshwater. (**C**) Abundance distribution pattern of 663 shared OTUs across soil (brown) and fresh water (blue) sites. X-Axis represents the average proportion of the shared OTUs in the complete soil and freshwater community and Y-axis represents the affiliation to soil and freshwater. A cutoff at 0.00025% is chosen as shared OTUs that represent more are spurious. Curve fitting was done with a generalized additive model (y ~ s(log(x))).

The vast majority of the OTUs were exclusive to either soil or freshwater. Only 6.3% of the OTUs (663) were shared between both habitat types (Fig. 2B) while 35.9% (3771) occured exclusively in freshwater and 57.8% (6081) exclusively in soil (Fig. 2A). Even though the fraction of shared OTUs was already low, the distribution of most of these shared OTUs showed strong preferences to either soil or freshwater indicating that the fraction of habitat generalists is considerably smaller (Fig. 2C; please refer also to Tables S2 and S3 for an overview on the presumably generalistic taxa, i.e. taxa with a relative read abundance between 25 and 75% in soil and in freshwater, respectively. For an overview of all shared OTUs see Table S4). It is noteworthy that the analysis of corresponding sites revealed an average of 20 ± 18 shared OTUs (min. 3, max. 87).

Even though only 6.3% of the OTUs were shared between soil and freshwater, their relative share of the OTU richness in individual samples was considerably higher reflecting a comparatively wide distribution of these OTUs: shared OTUs on average account for 14.6% of the OTUs in soil sites (min. 6.9%, max. 25.42%) and for 13.32% in freshwater sites (min. 5.25%, max. 32.74%). With respect to average relative read abundance, the importance of the shared OTUs is even higher representing 24.28% of the reads in soil sites (min. 3.52%, max. 56%) and 31.26% in freshwater sites (min. 1.13%, max. 68.06%).

The importance of highly abundant OTUs among those shared between both habitat types together with the slope of the curve in Fig. 2B indicate that in particular soil OTUs may be randomly dispersed to freshwaters (Fig. 2B green frame): most of the shared OTUs strongly dominate in soils (with low fractions of reads in freshwaters) indicating potential soil origin of these OTUs while considerably less OTUs showed the opposed pattern of high read fractions in freshwater and low fractions in soils (Fig. 2B yellow frame). Interestingly this tendency to specialize (with respect to habitat type) differed between OTUs with low and high read abundances: while many OTUs with low to moderate read abundances occurred in similar fractions in both habitat types, those with high read abundances strongly dominated in just one of the habitat types (Fig. 2C), i.e. may be considered potential habitat specialists (randomly dispersed to the other habitat type).

Irrespective of the presence of the shared OTUs in both habitat types, the richness of shared taxa within distinct samples is systematically affiliated with different taxonomic groups in soil and in freshwater (Fig. 1): In soil samples the majority of OTUs detected in both habitat types were affiliated with opisthokonts (especially fungi), followed by Ciliophora, Cercozoa and Stramenopiles. In contrast, in freshwater samples the majority of shared OTUs was affiliated with Chlorophyta, Cryptophyceae, Katablepharidaceae and Ciliophora.

Beyond this general pattern we also observed a systematic difference between rare and abundant OTUs: Shared OTUs with high abundances were predominantly affiliated with taxonomic groups considered to be dominant in the respective habitat, e.g. fungi and gliding taxa in soil, while shared OTUs with low abundances were affiliated to a wider (and more stochastic) selection of taxa (Fig. S4). For instance, in soils the relative contribution of opisthokonts (in particular fungi) to the fraction of shared OTUs was high within the abundant OTUs while the relative contribution of Chlorophyta and Chrysophyceae but also of Apicomplexa and Peronosporomycetes was higher within the rare OTUs (i.e. OTUs with low abundances). The contribution of Cercozoa and Ciliophora was similar for abundant and rare OTUs (Fig. S4).

In contrast, in freshwater the share of opisthokonts was high in the fraction of OTUs with low abundances but low within the abundant OTU. In contrast, Ciliophora, Oomycota and Chrysophyceae are more important in the fraction of OTUs with high read abundances. It is noteworthy that Apicomplexa and Cercozoa seem to play a minor role of the taxonomic overlap in freshwater (Fig. S4).

## Discussion

### Differential pattern of diversity between soil and freshwater

It is well known that morphological and molecular community analyses systematically deviate regarding the relative importance of distinct taxa. For instance, a higher relative abundance has been demonstrated for ciliates in molecular surveys which is due to different factors including copy number variation of the ribosomal genes^79^. Nevertheless, morphological as well as molecular surveys reveal corresponding trends in richness as long as the methodology within one study is consistent. For instance, community composition of soil clearly differed from that of freshwater in our study (Fig. 1). This is consistent with molecular^28,29,38,46,80^ and morphological surveys^24,81,82^.

Irrespective of a generally high community dissimilarities both for soil and for freshwater (which was more pronounced in soils), we found a higher richness in soils which is consistent with the literature (e.g.^14–17,24^). Interestingly, evenness of protists was generally higher in soil as compared to freshwater while it was the other way round for fungi. In other words the dominance of few protist taxa is more pronounced in freshwater lakes while dominance of few fungi is more pronounced in soils. This finding is noteworthy as it supports a differential role for community and ecosystem stability in soils and freshwater for protists and fungi with respect to the local extinction of distinct species (cf.^83–85^).

### Community overlap between aquatic and terrestrial habitats

It is well documented that soils and lakes host different protist communities as their environmental characteristics are fundamentally different^24,29,47,86^. However, as boundaries between habitats are diffuse, exchange of taxa and shared taxa between both compartments are proven^38,49,50^. For instance, Crump et al.^50^ showed that an arctic freshwater lake harbors 18% of the microbial eukaryotic upslope community and Graupner et al.^38^ demonstrated the non-permanent exchange of taxa between the compartments as a result of flooding. Here we show for a set comprising 30 sites that the number of shared microbial eukaryotic OTUs between soil and freshwater lakes is, however, very low (6.31%).

The small fraction of taxa occurring in both habitats indicate that communities presumably largely consists of taxa which are typical for either soil or freshwater and for which probably only few cells were dispersed to the other habitat type by chance. This view is supported by a strongly uneven share of most of these taxa to soil and freshwater communities (Fig. 2C). Among those OTUs which were found in both habitats in particular the most abundant ones were strongly unevenly distributed indicating that they are characteristic for one habitat type and just few cells may have been dispersed by chance. Only a few taxa (and interestingly predominantly taxa with low overall relative abundances) seem to be of similar importance in both habitat types and can thus presumably be considered as habitat generalists (Tables S2 and S3). For instance, within the taxa shared between both habitats we found sequences affiliated with taxa known to occur in soil and freshwater such as the ciliate *Microdiaphanosoma arcuatum*^87^, the ascomycete *Tetracladium maxilliforme*^88,89^ and the diatom *Fistulifera pelliculosa*^90,91^. In contrast, some other OTUs found within the shared fraction were previously known from only one habitat type, e.g. the OTUs related to the ascomycete *Podosphaera fusca*^92^ and the ciliate *Phialina salinarum*^93^. This is not necessarily contradictory to our results as sequence similarities of our OTUs to these latter species were often rather low and may not sufficiently resolve closely related species varying in their environmental demands^94^. Further, the resolution of the V9 region may be not appropriate for separating individual fungal taxa^95^ and therefore inferring information from the assigned taxa may be misleading.

Our data indicate that in particular taxa affiliated with Opisthokonta, Cercozoa and Apicomplexa may rather be specific for soils and their presence in freshwater samples is presumably largely due to random dispersal^96–98^. This is consistent with the study of Graupner et al.^38^ which demonstrated that despite an exchange of these taxa between soil and water, most of the exchanged taxa fail to establish in the new environment. As Opisthokonta (especially fungi), Apicomplexa and Cercozoa are highly abundant in terrestrial habitats^29,99–101^ the chance of random dispersal to freshwaters is high for these taxa.

In contrast, taxa assigned to Chlorophyta, Peronosporomycetes and Chrysophytceae may rather disperse from freshwater to soil habitats. This seems also conclusive as in particular Chlorophyta and Chrysophyceae are more abundant in freshwater than in soil^29,99^. This is possibly also true for Chytridiomycetes as their abundance and diversity in freshwater is slightly higher—again dispersal from freshwater to soil has been demonstrated^38^. Our results also indicate a predominant exchange of Peronosporomycetes from water to soil. For this taxon, however, published data indicate an exchange from soil to water^38,102^. Possibly, this hints to differential routes of dispersal for different taxa^94^ but data so far are inconclusive. Peronosporomycetes may nevertheless be an interesting taxon for further studies on habitat specificity and dispersal.

For Ciliophora the dominant direction of dispersal between the two habitat types is even less clear. Numerous ciliate OTUs occurred in both habitat types and these taxa made up for a similar share in freshwater and in soil communities^38,46,47,99,103,104^.

Overall, our data indicate that the direction of dispersal is predominantly from soil to freshwater (Fig. 2B): A majority of the shared OTUs occurred predominantly in soils with only low read numbers in freshwater. At first sight, this may seem to confirm the idea of soil protists as aqueous organisms^33^, with an aquatic origin which may therefore be able to cope with aquatic environments while freshwater protists lack an adaption to terrestrial habitats^105^. However, our data demonstrate that the vast majority of OTUs is habitat specific with only a very minor fraction capable of maintaining in both habitat types. Even for the fraction of shared OTUs our data indicate that the majority of taxa presumably is not established in both habitat types and that the presence of taxa in both habitats may largely be due to random dispersal rather than a broad niche adaptation^96,106^.

Nevertheless, despite the small number of shared OTUs they account for an integral part (up to ~ 68%) of the read abundances. This does not necessarily indicate a high abundance of generalistic taxa but may be rather due to a higher chance of random dispersal and subsequent random detectability of these taxa in both habitats. This view is supported by the high fraction of shared taxa with a strongly biased distribution towards either soil or freshwater and by the fact that particularly OTUs with high read abundances show such strongly biased distributions (Fig. 2C). Further, these latter taxa mostly belong to taxonomic groups considered either typical for soils (such as fungi) or freshwater (such as distinct algae).

Only some taxa with low to moderate read abundances show a rather uniform pattern across soil and fresh water, indicating that these may be true generalists without a distinct habitat preference (Table S3). For some of these taxa the presence in aquatic and terrestrial habiats was already shown as e.g. for *Cladosporium cladosporioides*^107^, *Gomphonema parvulum*^108^ and *Pythium capillosum*^109,110^, while other presumably generalistic taxa so far were known only from one habitat type (e.g. *Boeremia exigua* (terrestrial) and *Articulospora proliferata* (aquatic)).

As we cannot exclude the possibility that some of the OTUs in our data set represent inactive cells (e.g. resting stages, dead organisms) the number of shared OTUs may in fact be even smaller. Our results provide evidence that either the exchange of organisms is very low, the survival of these organisms in the other habitat type is low or both. However, we have to admit that the sampling depth of our study (as any such study) is restricted to the sampling volume. According to the sampling volume of several hundred milliliters of water and several grams of soil in our study we most likely have missed taxa which are very rare in a compartment (few individuals per liter of water / per gram of soil). That may explain why the overlap between corresponding soil and freshwater sites is very low and commends the general investigation of all freshwater sites and all soil sites. Thus, several of the taxa found to be habitat-specific may occur in the other habitat types at low abundance which may reflect, however, most likely random drift of some cells rather than true occurrence as an active member of the respective community. We also cannot exclude the possibility that some taxa were missing in either habitat due to spatial or seasonal variability in particular as soil communities differ over scales ranging from hectares to square millimeters, even when topography and texture are relatively uniform^20,84,111–113^ and samples were taken a few years apart. We are aware that soil and freshwater campaigns did not take place in the same year. Still, Gilbert et al. and Bock et al. showed that microbial communities show repeatable seasonal patterns and Shade et al. showed that microbial communities can stay relatively stable over time and as we sampled during mid summer we expect that the abundances may changed, but the mere presence of OTUs should be stable^9,114,115^. The intention of our study was not to link active interacting communities but rather to reflect differences and commonalities between soil and aquatic communities on a comparable spatial (geographic) scale. We therefore consider the temporal difference to be neutral (if not advantageous) as the temporal and logistic separation decreases the chance of (natural and artificial) cross contamination between aquatic and terrestrial samples while taxon coverage may be rather stable due to resting stages in the seedbank of soils^9,41,42^.

However, in future studies the inclusion of freshwater sediments seems reasonable as conditions between soil and aquatic sediments may be more similar and soil organisms may deposit to and dwell in freshwater sediments even if they cannot compete in the pelagial. Further, sampling over a long time period could provide valuable information about the long-time establishment of exchanged species.

## Conclusions

Our study showed that the community in soil and freshwater is fundamentally different and that co-occurring OTUs are rare. In addition, abundant shared OTUs are rather affiliated with one habitat type and most likely dispersed to the other habitat by chance. Only few rare shared OTUs may represent true habitat generalists. This gives evidence , that soil and freshwater communities are rather closed communities and that an establishment of taxa from the respective other habitat type is unlikely even though inoculation occurs and, in particular freshwater habitats seem regularly to be inoculated by individual OTUs originating from soil.

Further, soil habitats show a significant higher OTU richness and higher diversity compared to freshwater habitats, which is also reflected by a higher community dissimilarity compared to freshwater habitats. However, true diversity was similar for fungi as richness was higher in soils but evenness was higher in freshwater.

## Supplementary information

Supplementary Information.
